# Ethical Issues in Measuring Biomarkers in Children’s Environmental Health

**DOI:** 10.1289/ehp.0800480

**Published:** 2009-05-06

**Authors:** Peter D. Sly, Brenda Eskenazi, Jenny Pronczuk, Radim Šrám, Fernando Diaz-Barriga, Diego Gonzalez Machin, David O. Carpenter, Simona Surdu, Eric M. Meslin

**Affiliations:** 1 WHO Collaborating Centre for Research on Children’s Environmental Health, Curtin University of Technology, Perth, Australia; 2 Centre for Child Health Research, University of Western Australia, Perth, Australia; 3 Center for Children’s Environmental Health Research, University of California at Berkeley, Berkeley, California, USA; 4 Public Health and Environment, World Health Organization, Geneva, Switzerland; 5 Institute of Experimental Medicine, Prague, Czech Republic; 6 WHO Collaborating Centre for Health Risk Assessment and Children’s Environmental Health, Universidad Autónoma de San Luis Potosí, San Luis Potosí, Mexico; 7 Regional Advisor in Toxicology, World Health Organization/Pan American Health Organization Country Office, Brasilia, Brazil; 8 Institute for Health and the Environment, University at Albany, The State University of New York, Albany, New York, USA; 9 Public Health and Environment, World Health Organization/Interregional Research Unit, Research Triangle Park, North Carolina, USA; 10 Indiana University Center for Bioethics, Indianapolis, Indiana, USA

**Keywords:** biobanks, biomarkers, children, environmental exposure, genetics, infants, informed consent, research ethics

## Abstract

**Background:**

Studying the impact of environmental exposures is important in children because they are more vulnerable to adverse effects on growth, development, and health. Assessing exposure in children is difficult, and measuring biomarkers is potentially useful. Research measuring biomarkers in children raises a number of ethical issues, some of which relate to children as research subjects and some of which are specific to biomarker research.

**Objective:**

As an international group with experience in pediatric research, biomarkers, and the ethics of research in children, we highlight the ethical issues of undertaking biomarker research in children in these environments.

**Discussion:**

Significant issues include undertaking research in vulnerable communities, especially in developing countries; managing community expectations; obtaining appropriate consent to conduct the research; the potential conflicts of obtaining permission from an ethics review board in an economically developed country to perform research in a community that may have different cultural values; returning research results to participants and communities when the researchers are uncertain of how to interpret the results; and the conflicting ethical obligations of maintaining participant confidentiality when information about harm or illegal activities mandate reporting to authorities.

**Conclusion:**

None of these challenges are insurmountable and all deserve discussion. Pediatric biomarker research is necessary for advancing child health.

Studying the health impacts of environmental exposures on children is assuming increasing importance. There is a general recognition that children are more vulnerable to such exposures by virtue of the higher doses they receive and their increased susceptibility (Sly and Flack 2008). Children’s increased susceptibility is a consequence of the higher exposure and uptake, relative immaturity of metabolic and excretory pathways, and incomplete development of target organs [World Health Organization (WHO) 2006]. Children with the null mutation of glutathione *S*-transferase enzymes are at increased risk of asthma when exposed to traffic-related pollutants (Romieu et al. 2006), and children with a specific mutation in paraoxonase-1 are more susceptible to the adverse effects of exposure to pesticides (Holland et al. 2006). However, the fact that an individual child may be at increased risk from adverse effects from environmental exposures does not guarantee that the individual child will develop the adverse effect.

The range of environmental exposures affecting children’s health is expanding with increasing urbanization, especially in developing and emerging countries. Children living in mega-cities and heavily industrialized cities are exposed to levels of air pollutants far higher than those of their counterparts in many developed countries (Mucha et al. 2006; Romieu et al. 2002). Children are likely to participate in recycling activities involving recovering saleable but toxic components such as lead from batteries and components from discarded electronic equipment and computers (Holdren et al. 2008). The rapid introduction of new chemicals in many countries, especially where the health effects have not been fully evaluated, are likely to pose a problem for children (e.g., polybrominated flame retardants used in children’s sleepwear), especially in developed countries (Holdren et al. 2008).

Assessing exposures in children is more difficult than it is in adults (Needham and Sexton 2000). One approach to exposure assessment in children is to measure biomarkers of exposure and biomarkers of the effects of that exposure. A biomarker of exposure is a xenobiotic substance or its metabolite that is measured in the body and can be related to an environmental exposure; examples include measuring cotinine in urine or blood to give an indication of exposure to nicotine in tobacco smoke and measuring benzene metabolites in urine to give an indication to exposure to traffic-related pollution. Where a metabolite is measured, the child’s ability to metabolize the pollutant as well as the exposure must be considered. A biomarker of effect measures direct or indirect consequences of the environmental exposure and incorporates the child’s ability to defend against the exposure—for example, exposure to traffic-related pollutants results in the generation of reactive oxygen species in the lungs and oxidative stress if the antioxidant defenses in the lungs are overwhelmed (WHO 2001). Oxidative stress in the lungs can result in tissue damage from diverse mechanisms, including lipid peroxidation, protein halogenation, and DNA oxidation. By-products of these processes [e.g., 8-isoprostone and malondialdehyde (Romieu et al. 2008) from lipid peroxidation, 3-chlorotyrosine from protein halogenation (Harwood et al. 2006), and 8-oxodeoxyguanosine from DNA oxidation (Cooke et al. 2006)] can be measured in various body compartments including the lungs (sputum, bronchoalveolar lavage), blood, and urine. Clinical effects on the child [e.g., lower lung function in children exposed to tobacco smoke, especially during pregnancy (Stick et al. 1996; Vork et al. 2007), or exposed to traffic-related pollution (Gauderman et al. 2004), and delayed neurodevelopment in children exposed to neurotoxicants (Sly and Flack 2008)] can also be considered to be biomarkers of effect.

In some circumstances it is also possible to measure biomarkers of susceptibility to environmental exposures. This concept is best understood in the field of cancer, where specific chromosomal markers are related to disease susceptibility and prognosis (Duncan 2004; Malkin 2004), but does occur in non-cancer fields, for example, red cell glucose-6-phosphate dehydrogenase deficiency.

Biomarkers can be used in intervention studies to determine the efficacy of environmental interventions. Biomass fuel burning is an enormous problem globally, and fuels used for domestic cooking or heating emit a complex mixture of organic compounds and gasses that can include carbon monoxide, oxides of nitrogen and sulfur, aldehydes, polyaromatic hydrocarbons (PAHs), volatile organic compounds, chlorinated dioxins, and particulate matter when burned. Torres-Dorsal et al. (2008) studied the efficacy of improved stoves and housing on exposure of children to the emissions from biomass fuel combustion. They demonstrated a reduction of exposure as the serum levels of 1-hydroxypyrene (biomarker of PAH exposure) and carboxyhemoglobin (biomarker of CO exposure) declined and a reduction of effects via a reduction in DNA damaged, assessed using a comet assay (Torres-Dorsal et al. 2008).

## Ethical Aspects of Research Involving Children

The study of biomarkers in children raises a number of special ethical considerations related to the collection and storage of specimens, consent, and how to convey information about risk, especially where the level of scientific knowledge is inadequate to quantify that risk (Eskenazi et al. 2005). Many of these issues are similar to those involving adults (Caulfield et al. 2007; Evans and Meslin 2006; Helft et al. 2007; Malkin 2004), but other issues may be unique to children (Neidich et al. 2008). In this commentary we concentrate on issues specifically related to children rather than general issues.

Pediatric biomarker research is especially worthy of special ethical scrutiny, because it invokes issues arising in pediatric research generally coupled with those of environmental health—biobanking and genetics research more specifically. Moreover, when these types of studies are carried out in economically developing countries, further ethical issues emerge. Before turning to the particular issues in pediatric biomarker studies, we first discuss the general ethical issues in pediatric research.

The ethics of research involving children has a long and profound history including important debates in the early 1960s that made compelling arguments for and against (McCormick 1976; Ramsey 1976). With the revelations of unethical research involving medicines in pregnant women, children, and fetuses in the 1960s (Beecher 1966), landmark legislation was passed in 1974 by the U.S. Congress which, among other actions, established the National Commission for the Protection of Human Subjects of Biomedical and Behavioral Research (1979). Several commission reports, including Research on the Fetus (National Commission for the Protection of Human Subjects of Biomedical and Behavioral Research 1975), Research Involving Children (National Commission for the Protection of Human Subjects of Biomedical and Behavioral Research 1977), and the *Belmont Report* (National Commission for the Protection of Human Subjects of Biomedical and Behavioral Research 1979), provided the ethical foundations for what would become the U.S. regulatory mechanism to protect human subjects from harm, including the Common Rule (45 CFR 46, Subpart A; Department of Health and Human Services 2005), the relevant FDA regulations (21 CFR 50/56; FDA 1998), and additional provisions for the protection of other vulnerable subjects including children (45 CFR 46, Subpart D; Department of Health and Human Services 2005).

Statements on the ethical conduct of research issued by various national and professional bodies generally include a section outlining the special requirements for including children in research [Canadian Institutes for Health Research 2007; Council for International Organizations of Medical Sciences (CIOMS) 1993; Meslin and Johnson 2008; National Health and Medical Research Council Australia 2007; National Institutes of Health 1998; World Medical Association 2008]. Common to all of these guidelines are three general protections:

- Sound justification—the presumption that children should not be included in research unless there is a compelling reason to do so. This has the potential to exclude children from important research. In an attempt to address this issue, a major shift in U.S. regulatory policy occurred in 1993. Rather than excluding women and children from research, the National Institutes of Health (NIH) made clear its commitment to requiring that women be included in trials unless there was a reason not to in the 1993 NIH Reauthorization Act (NIH 1993).
- Informed consent—the basis for this assessment is sometimes found in the bioethical principle of respect for persons described in the *Belmont Report* (National Commission for the Protection of Human Subjects of Biomedical and Behavioral Research 1979) or respect for autonomy (Beauchamp and Childress 2005). Both principles understand that only adults are presumed to have the capacity to act autonomously, whereas children are not presumed to have this capacity. In general, consent is given on their behalf by parents or legal guardians, usually supplemented by the positive affirmation (or assent) of the child where possible (De Lourdes Levy et al. 2003).
- Prior ethics review.

An expanded discussion of these principles and the impact on research in children can be found in the Supplemental Material (doi:10.1289/ehp.0800480.S1).

### Local versus global ethical standards

Common to most guidelines that address research involving children is a recognition that certain bioethical principles ought to guide decision making. Some of these, such as respect for persons, beneficence, and justice, have provided the moral foundation for the protections now commonly adopted by ethics review committees in the United States and elsewhere. Although these principles reflect the high value Western societies place on individual autonomy, it is important to remember that this is not the only way in which humans interact and responsibilities are conceptualized in various non-Western societies and in some indigenous societies within Western countries. These factors also play a role in determining the ethical acceptability of conducting research on children in those communities. However, other principles have been suggested and defended, including solidarity and community participation (CIOMS 1993; Emanuel and Weijer 2005; Needham and Sexton 2000).

The national and international statements governing research in economically developed countries generally do not recognize the importance of local community values, either within a country or between countries. Research-funding agencies generally require researchers to obtain ethical approval for the research to be conducted in the jurisdiction from which the funding has been obtained, regardless of where the research is to be conducted. Problems may arise when funding is obtained in an economically developed country for research to be conducted in an economically developing country. This issue has been highlighted in the area of clinical trials (Sewankambo and Ijsselmuiden 2008; Shaffer et al. 2006; Shapiro and Meslin 2001), but applies to other area of research as well (Andanda 2008; Angell 1997; Anonymous 2007; Sharp and Zigas 2002). Clearly, researchers should take the local cultural issues into consideration (Meslin 2009; Widdows 2007), and ethics review boards in economically developed counties should give preference to the local cultural values and differences in laws, for example, laws governing privacy of participants and security of data (Sidle et al. 2006).

There is a particular problem regarding obtaining informed consent when collaborations are established in communities where the cultural practice is that consent is provided by the leaders of the community, not by individuals. This practice is obviously contrary to the basic assumptions behind our Western concept of informed consent. However, to ignore the culture of a community is both inappropriate and will almost certainly doom any prospect of collaboration. The best practice that bridges cultures is for the investigators to first obtain approval for the study from the community leaders or whoever is the traditional gatekeeper for the community. If that is successful, then the investigators can approach the individuals to obtain their agreement to participate following usual procedures. However, even if this sequence is followed, there are justifiable concerns: Often the community leaders will basically order the members to participate, and the individuals feel that they have no alternative but to agree with that decision. Although this is not what is meant by informed consent and may not easily pass institutional ethics review boards, the alternative is that no studies can be done with these communities. This is equally unacceptable and discriminatory.

## Ethical Issues in Biomarker Research in Children

The study of biomarkers in children raises several ethical issues. Many of these arise in research involving children generally, as described above; others are more particularly related to the collection and storage of specimens, consent, and how to convey information about risk, especially where the level of scientific knowledge is inadequate to quantify that risk (Eskenazi et al. 2005). Many of these issues are similar to those involving adults (Caulfield et al. 2007; Evans and Meslin 2006; Helft et al. 2007), but other issues may be unique to children (Neidich et al. 2008). We highlight two of these issues.

### Community expectations

Research using biomarkers is carried out to learn about what a particular biomarker can tell the researchers about environmental exposures, susceptibility, or risks for adverse health outcomes from those exposures. Thus, such research is generally undertaken in exposed communities, which may raise expectations within the community that the study will *per se* improve the situation in the community (Eskenazi et al. 2005). However, the reality is that, in many cases, the risks are poorly understood and the primary purpose of conducting the study is to clarify the associations between exposures and health consequences and the role of biomarkers in understanding these associations. As pointed out by Eskenazi et al. (2005), understanding the community expectations and developing a communication strategy before starting the research is an important part of biomarker research. These issues are highlighted in two case reports outlining studies in children exposed to pesticides [Children’s Environmental Exposure Research Study (CHEERS)] or lead (Kennedy Krieger), which are included in the Supplemental Material (doi:10.1289/ehp.0800480.S1). These cases provide valuable lessons for conducting environmental health research in a community.

What can a community reasonably expect from participating in biomarker research? There is no simple answer to this question, because community expectations from research often vary widely. Thiessen et al. (2007) undertook an assessment of what individuals in a low-income community in Uganda perceived as benefits and harms of population-based human immunodeficiency virus/sexually transmitted disease (HIV/STD) research in their community. Those who participated and those who declined participation equally believed that there were benefits to participation. Those who had declined to participate, frequently because they believed that they were being harmed, believed that the research would benefit the local economy, improve health worker knowledge about HIV/STD, and improve health of local residents in the future (Thiessen et al. 2007). Improved knowledge about the health risks from environmental exposures can be of benefit to communities in which that research is undertaken. However, it is important to clearly communicate to communities what they can and cannot expect from the research. Models for engaging communities on this topic exist, one of which is referred to as a prior agreement, in which communities and researchers negotiate in advance of a study what benefits may be expected or provided at the end of the study (Page 2004).

### Return of research results

Although there may be a general expectation that research results will be communicated to the study participants and to the community, both practice and ethical guidance vary widely. Some argue that only under exceptionally rare circumstances should research results be returned to study participants—specifically, those instances in which a known harm can be avoided by referring a person to a physician and where the test result is valid (Wolf and Lo 2004). This argument is especially germane to genetic information when the results were obtained from a research laboratory and not a clinical genetics facility whose focus is on providing diagnostic quality data to patients. In the latter case, providing results of clinical tests that have a direct bearing on the health of an individual is relatively straightforward, and few would argue against this approach. However, the further the data stray from having a direct effect on the health of the individual, the more opinion varies. Such issues have recently been highlighted in discussions of predictive genetic testing in young people (Duncan 2004; Malkin 2004; Quaid et al. 2004). Duncan (2004) recently reviewed the ethics of such predictive genetic testing in children and reported that the default position in guidelines published by the international Huntington Association and the Clinical Genetics Society is to refuse testing for young people < 18 years of age. This opinion is based on the view that the psychosocial harm involved in testing outweighs any benefits in the absence of preventative treatments. These arguments could also be applied to much biomarker research, especially in children. However, this would result in children being excluded from studies on environmental exposure where biomarkers are measured.

Much current biomarker research is conducted to understand what, if any, role biomarkers can have in understanding the health consequences of environmental exposures. In these cases, almost by definition, there is a lack of knowledge about how the level of or the presence of a particular biomarker translates into disease risk for an individual, and one could well argue that communicating information that the researcher cannot understand to research participants and communities is an abrogation of the researcher’s responsibility. To tell a mother that her child has a detectable level of a certain biomarker without also being able to tell her what that means in terms of disease risk may do more harm than good.

The opposing point of view, that participants have the right to know, would lead researchers to provide results even in the face of uncertainty of their significance. Needham and Sexton (2000) express this in terms of an explicit or implicit social contract between the researchers and the participants that stipulates that the researchers will interpret the health significance of measured exposure levels. This would also extend to biomarkers. Thus, where the data cannot be interpreted in terms of health significance, should they be communicated to participants?

This dilemma of providing results is illustrated in the Center for the Health Assessment of Mothers and Children of Salinas (CHAMACOS) study, a longitudinal birth cohort study of pesticide exposure and health consequences to children from primarily poor Latino farmworker families living in California (CHAMACOS 2009). This study is a community-based participatory research project that receives advice from a community advisory board on research issues. As part of the exposure assessment, urine from women and children was analyzed for pesticide metabolites. When the CHAMACOS study began in 1999, there were no reference data, and the health effects associated with these levels was unknown. Consequently, the researchers, in consultation with the community advisory group, stated clearly in the consent forms that no individual results would be disclosed. However, in 2003, U.S. National Health Survey data became available, allowing for the levels in the CHAMACOS study participants to be compared with a national reference. Members of the study’s community advisory board felt strongly that the results should then be disclosed to participants, although the health consequences were still unknown. With ethics review board approval, consent forms were changed, and results were then provided in person to parents who requested them. No adverse consequences of this change in policy have been noted.

### Conflicting ethical obligations

Community-based research, especially when conducted in vulnerable populations, has the risk of creating conflicting ethical obligations for researchers. For example, protecting against unauthorized access to confidential information about research participants is a key focus of ethics review boards, researchers, and research participants. However, legal obligations, including mandatory reporting of child abuse and illegal activities, can put researchers in a difficult position if such activities are revealed during the research. In the United States, the new Genetic Information and Nondiscrimination Act (2008) provides protections, and certificates of confidentiality afford specific protections against certain compelled disclosures, but neither has been tested in cases of pediatric biomarker research. Participants might expect that researchers will not reveal details of private conversations between children and researchers to the child’s parents without the express consent of the child, or about children or family members to authorities. What do researchers do about information that children are being abused, engaging in risky sexual practices, or abusing substances if these are revealed during a confidential interview or observation? One option is to establish an external reference group that will consider the appropriate action to be taken if such disclosures are made. If the existence and purpose of the reference group is made known to the research participants and their parents before their consent to participate, the ethical dilemmas can be reduced. In the CHAMACOS study, the consent explicitly states that “if we see or hear something that would endanger you, your child, or others, we may discuss it with you, if possible, or seek help. If we learn that your child may be harmed, we may have to report this to Child Protective Services.” In this way, it is clearly stated that the child’s safety is paramount and that in cases where the child is considered to be at risk, confidentiality will be broken to protect the child.

### Informed consent

The principle underlying informed consent—personal autonomy—presumes that individuals will be given sufficient information to make an informed choice about their participation in research. However, we wonder whether informed consent can be obtained from research participants in biomarker research if the researchers themselves do not fully understand the relationship between environmental exposures, biomarkers, and health consequence. A similar problem arises for biobanks when researchers ask potential participants to donate DNA or tissue that may not be used for many years in the future and for which no specific research protocol has been designed. As with one-time consent for biobanks (Caulfield et al. 2007), the challenge is whether research participants have sufficient information to decide. We think that if the research proposal adequately discloses the lack of the researcher’s knowledge and describes the research purpose to the best of the researcher’s ability, subjects and communities can make an appropriate decision about participation (Schulte et al. 1997).

The issue of competent minors also comes into determining who is able to give consent for research procedures. In some jurisdictions, including Australia, legally recognized consent to participate in research can be given only by individuals ≥ 18 years of age. Parents or legal guardians are obliged to give consent for children under this age to participate. However, legal minors can consent to the provision or withholding of medical treatments if, in the view of the treating physician, they are competent to make such a decision. Where research involves the provision of medical treatment, the situation is less clear (Dixon-Woods et al. 2006).

In the absence of legal consent from children, the practice adopted in most countries is to obtain assent from the child to participate in the research provided a parent or guardian provides consent. The assent of a child cannot override a parental decision to withhold consent, but neither should the consent of a parent or guardian override a child’s decision not to assent to the research (De Lourdes Levy et al. 2003). Researchers should respect the wishes of the child about not participating in research. The issue of when a child is competent to provide assent is not a simple one. A reasonable practice is to provide children with simple information sheets written in age-appropriate language that sets out exactly what will be expected of them and what will happen to them in the research. This information should also explain in simple and explicit language that the child has the right to change his or her mind at any time and withdraw from the research without fear of consequence.

Another issue frequently faced in longitudinal studies, especially those in which biological samples or biomarkers have been collected and stored, is obtaining consent from participants once they gain the age of legal majority (18 years of age in most jurisdictions). The generally held view is that researchers must contact participants where consent was given by a parent or guardian and obtain consent for the continued use of data and biological specimens. Difficulty in contacting participants is not usually an acceptable excuse for not doing so. Indeed, U.S. research regulations expressly prohibit the inconvenience of obtaining consent as an acceptable justification for not trying. A more rigid criterion, impracticability, is used, which refers to a level of difficulty that would make it virtually impossible to carry out the study. That is, if obtaining consent, including recontacting those who gave diagnostic samples for use in research, makes it extremely difficult to carry out the study, then ethics committees are permitted to waive the consent requirement.

The legal issues involved in determining ownership and intellectual property rights over stored biological samples are complex (Andanda 2008). The ethical use of biological material collected in developing countries for use in economically developed countries is more complex (Upshur et al. 2007). Ethics review boards need to satisfy themselves that appropriate consent was obtained from the research subjects, using the procedure deemed appropriate by the local community and local ethical review board (Upshur et al. 2007). This may involve viewing in detail the translations of information sheets or informed consent documents and/or letters of approval from the relevant local authorities.

### Research into housing-related health hazards and pesticide exposure in children

Although the primary purpose of housing is to provide a safe haven for normal living, there is an increasing recognition of the hazards that may exist in the indoor environment. A healthy and safe physical and psychosocial home environment is essential for the normal development of children, especially during the preschool years. Poor-quality housing and hazards found within the house can affect children’s health. In developing countries, the major hazards may include poor construction related to lack of high-quality building materials and skills; poor location on contaminated or disaster-prone sites; lack of basic services such as clean water, sanitation, and adequate waste disposal facilities; chronic infestation with rodents and other disease vectors; the use of biomass or solid fuel for cooking or heating; and overcrowding. In industrialized countries where the provision of basic services is likely to be adequate, hazards may include poor housing design or use of unhealthy building materials; poor heating or ventilation systems encouraging high loads of bioaerosols and triggering or inducing chronic diseases such as asthma; and exposure to radon or electromagnetic radiation (Pronczuk and Surdu 2008).

Along with the increasing recognition of the health hazards associated with housing has come an increase in research into the health effects of housing environments. Two relatively recent and well-publicized controversial events—namely, CHEERS and the 2001 case of Grimes *v.* Kennedy Krieger Institute [see Supplemental Material (doi:10.1289/ehp.0800480.S1)]—have highlighted ethical concerns with this type of research involving children. Debate has continued, especially in the area of research involving exposure of children to pesticides (Needleman et al. 2005; Resnik and Portier 2005), with Resnik and Portier contending that the benefits of intentionally exposing humans to pesticides outweigh the risks of doing so. (They deliberately avoided the issue of testing in research involving exposure of children.) This argument was not accepted by a group of prominent investigators, who argued that the increased susceptibility of children to pesticides precluded the extrapolation of adult data to the pediatric age range. They also pointed out that it would be extremely difficult to adequately power an exposure study to detect effects that would harm 1 child in 1,000 (Needleman et al. 2005). In addition, the arguments advanced by Resnik and Portier (2005) do not address the issues of research involving children, as discussed above.

A U.S. National Academy of Sciences committee report, *Ethical Considerations for Housing-Related Health Hazards Involving Children* (Lo and O’Connell 2005), highlighted the ethical issues that are more common in housing-related research than in other biomedical research, including the following:

- The research may intrude into the privacy of residents.
- The research is generally community based and frequently involves community concerns about issues related to local housing.
- Housing hazard research is more likely to target low-income families who live in poor-quality housing; thus, children of low-income families are specifically targeted.
- Concerns are likely to remain about hazards that persist after the research has been completed.
- Economic and educational disadvantage and limited literacy may place low-income parents at a disadvantage in the informed consent process.
- Financial or other material incentives may present undue influences for parents in the decision to allow their children to participate in such research projects.

Readers interested in these issues are invited to consider the two case reports incorporated in the Supplemental Material (doi:10.1289/ehp.0800480.S1).

### The National Children’s Study

After many years of planning and preliminary studies, the U.S. National Children’s Study has been launched (National Children’s Study 2009). This study aims to include 100,000 children (with their families) from birth (or earlier) through 21 years of age and will include “a thorough history of exposures, biological samples, and health outcomes will be obtained from pregnancy onwards, allowing for comprehensive statistically powerful analyses of the link between a wide range of exposures and genetic factors with child health and development” (National Children’s Study 2009). Much preparation has gone into the study, including a through assessment of the requirements for biological monitoring at different life stages (Barr et al. 2005), the methodologic and logistic issues involved in conducting longitudinal birth cohort studies (Eskanazi et al. 2005), and the burden imposed on the family (Wagener 2003). This thorough preparation gives considerable comfort that this massive study will be conducted with due consideration of the ethical issues in research involving biobanks and biomarkers in children.

## Summary

Biomarker research in children poses some particular ethical problems, especially with regard to obtaining appropriate consent and deciding what information results can be fed back to the study participants and the community. Developing appropriate strategies for understanding and managing community expectations is important in all research, but especially for biomarker research involving children. An additional challenge is presented when this research is undertaken in countries where values, customs, and ethical guidelines are different from those countries where studies have been ongoing for several decades and a large body of experience has been collected. None of these challenges are insurmountable. Pediatric biomarker research is necessary for advancing child health.
